# ‘Two‐floret spikelet’ as a novel resource has the potential to increase rice yield

**DOI:** 10.1111/pbi.12849

**Published:** 2017-11-07

**Authors:** Deyong Ren, Haiping Yu, Yuchun Rao, Qiankun Xu, Tingting Zhou, Jiang Hu, Yu Zhang, Guangheng Zhang, Li Zhu, Zhenyu Gao, Guang Chen, Longbiao Guo, Dali Zeng, Qian Qian

**Affiliations:** ^1^ State Key Laboratory of Rice Biology China National Rice Research Institute Hangzhou China; ^2^ College of Chemistry and Life Sciences Zhejiang Normal University Jinhua China

**Keywords:** double floret, spikelet determinacy and indeterminacy, multi‐florets spikelet, grain number per panicle, rice breeding

Yield in rice (*Oryza sativa*) is determined by three major components: panicle number per plant, grain weight and grain/spikelet number per panicle (Zhou *et al*., 2015). Grain number per panicle is one of the main targets and mainly results from the number of spikelets. Traditionally, rice breeders have focused on the improvement of spikelet number per panicle and rarely focused on the number of florets because a normal rice spikelet has one fertile floret and produces one seed. In grass, the spikelet comprises one to 40 florets depending on the species and shows determinacy or indeterminacy. In rice (*O. sativa*) with a determinate spikelet, the spikelet meristems produced the fixed floral meristems, resulting in the formation of one floret. In wheat (*Triticum aestivum*) with an indeterminate spikelet, the spikelet meristems produced the variable floral meristems, resulting in the formation of more florets. How to further increase rice yield? If the number of florets in a spikelet could be increased, it may be a new method for rice high production. In our study, we characterized two allelic mutants with two florets within a single spikelet, *double floret1*‐*1* (*df1‐1*) and *df1‐2*. We next focused on the *df1‐1* mutant to investigate the regulation of floret number in rice, and this provided a new perspective for increasing grain number per panicle and yield.

**Figure 1:**
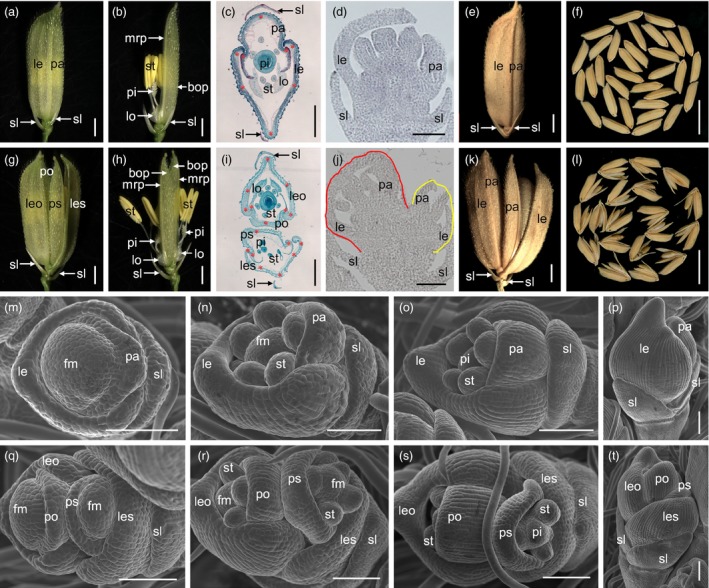
Phenotypes of spikelets in the wild type and the *df1‐1* mutant. (a–b) wild‐type spikelet. (c) Transverse section of the wild‐type spikelet. (d) Longitudinal section of the wild‐type spikelet. (e–f) Wild‐type seed. (g–h) *df1‐1‐*mutant spikelet. (i) Transverse section of the *df1‐1‐*mutant spikelet. (j) Longitudinal section of the *df1‐1‐*mutant spikelet. (k–l) *df1‐1‐*mutant seed. (m–t) Early spikelet development in the wild‐type and the *df1‐1* mutant. (m–p) Wild‐type spikelet. (m) Sp4, (n) Sp5‐6, (o) Sp7, (p) Sp8. (q‐t) *df1‐*mutant spikelet. (q) Sp4, (r) Sp5‐6, (s) Sp7, (t) Sp8. sl, sterile lemma; le, lemma; pa, palea; leo, lemma of the original floret; les, lemma of the secondary floret; po, palea of the original floret; ps, palea of the secondary floret; bop, body of the palea; mrp, marginal regions of the palea; lo, lodicule; st, stamen; pi, pistil; fm, floral meristem. Red stars indicate vascular bundles. Regions surrounded by red and yellow lines indicate different florets in (j). Bars = 1000 μm in (a), (b), (g), and (h); 100 μm in (c), (i) and (m–t); 100 μm in (d) and (j); 1 cm in (f) and (l).

The wild‐type rice spikelet has one fertile floret that is flanked by one pair of glumes, which are generated from the spikelet meristem, and one floret per spikelet is strictly regulated in the *Oryza* genus (Figure 1a–d). The floret comprises the lemma, palea, lodicule, stamen and pistil (Figure 1a–d).

The 15%–20% spikelets developed two florets inside one pair of sterile lemma which randomly distributed in the *df1‐1* mutant (Figure 1g–h). Rarely, three florets were also observed (Figure 1c). In the *df1*‐*1* mutant, we observed four whorls of floral organs within each single floret (Figure 1g–j). Each lemma and palea had a similar histological texture and vascular bundles as the wild type, respectively (Figure 1c,i). To confirm the identity of the organs in each single floret, we investigated the expressions of *OsMADS1*,* OsMADS14*,* OsMADS15*,* OsMADS6* and *DL* responsible for the lemma and/or palea identity. No differences in gene expression were found within each single spikelet between the wild type and *df1*‐*1* mutant. We next investigated the expression of the *OSH1* gene, which is essential for floral meristem activity. *OSH1* showed a much higher expression in the young panicles of the *df1* mutant. These results suggested that each floret in the *df1*‐*1* mutant has a normal lemma and palea, and two florets form within a single spikelet. At maturity, the seed setting rate, grain size and weight of the normal and original florets in the *df1*‐*1* mutant were comparable with those in the wild type, respectively. The seed setting rate of the secondary florets was lower, and the grain size and weight were variable in the *df1*‐*1* mutant. These results suggested that *DF1* has the potential for increasing the grain number per panicle and yield.

We next investigate early spikelet development. Compared with the wild type, we found two pairs of the lemma and palea in a single *df1*‐*1* spikelet at the Sp4 stage, which were generated from different floral meristems (Figure 1m,q). At the Sp5 and Sp6 stages, two florets within a single spikelet developed normal stamen primordia which exhibited a similar developmental process with that of the wild‐type floret (Figure 1c,i,n,r). At the Sp7 stage, with formation of the pistil, the stamen primordia of the original floret differentiated into quadrangular anthers, whereas the secondary floret displayed hemispherical stamen primordial (Figure 1o,s). At the Sp8 stage, two apparent rudiments of florets were observed within the single spikelet (Figure 1d,j,p,t). Taken together, the formation of two florets in a single spikelet revealed that the two floret meristems were generated from one spikelet meristem.

Then, we detected the expression of several marker genes by *in situ* hybridization. *OsMADS6* expression is uniformly found in the floral meristem of the wild‐type spikelet. We detected two independent *OsMADS6* signals within a single spikelet in the *df1*‐*1* mutant at the Sp4 stage. During stages Sp5 to Sp8, *OsMADS6* expression was strongly visible in two mrps of the wild‐type floret. In the *df1*‐*1* mutant, their signals were induced in four mrps of two florets in a single spikelet. At stages Sp4‐Sp8, *DL* was strongly expressed in the lemma of the wild type. However, *DL* signals were observed in the lemma of the secondary floret in addition to the lemma of the original floret. *OSH1* is the marker gene for indeterminate meristems, and abundant *OSH1* transcripts were observed throughout the early floral meristem in the wild‐type floret. Interestingly, at the Sp4‐Sp6 stage, two strong, independent *OSH1* signals were visible inside one pair of sterile lemma in the *df1*‐*1* mutant, implying that two floret meristems were present in a spikelet. In the wild‐type spikelet, *G1* signal was found in a palea. Two independent *G1* signals were detected inside a pair of sterile lemma in the *df1‐1‐*mutant spikelet, implying that two palea were initiated in the single spikelet. These findings revealed that the *df1*‐*1‐*mutant developed two floral meristems from one spikelet meristem and formed two independent florets in a single spikelet.

The *DF1* locus was narrowed to a 56‐kb region. A single‐nucleotide substitution from G to T in the *df1‐1* mutant and C to T in the *df1‐2* mutant was found within a predicted lipase gene (*Os01g0900400*). The complementation test showed that the *df1‐1* phenotypes were rescued, and the Cas9‐*DF1* mutant produced two florets and seeds, resembling that of the *df1* mutants. These results confirmed that *DF1* was *Os01g0900400*. Strong GUS signals from pro*DF1*‐GUS and *DF1* transcripts were specifically detected in the spikelets and panicles. *In situ* hybridization revealed that *DF1* transcripts were strongly expressed in the spikelet meristem, floral meristem, floral organs. Analysis of enzyme activity revealed that purified DF1 protein had lipase activity (3.85 U/mg protein), validating that *DF1* encodes a lipase.

Here, the *df1‐1* spikelets produced two fertile florets, suggesting that the spikelet determinacy was lost. In the *mfs1*,* tob1* and *snb* mutants, and *snb*/*osids1* double mutant, some spikelets developed an additional lemma‐like organ, implying that these genes regulate the spikelet meristem determinacy and the timing of the transformation from the spikelet meristem to the terminal floral meristem (Lee and An, 2011; Lee *et al*., 2006; Ren *et al*., 2013; Tanaka *et al*., 2012). Unlike the *df1‐1* mutant, extra florets of *mfs1*,* tob*,* snb* and *snb*+*osids1* spikelets bore a vestigial secondary floret that only possessed a lemma‐like organ, and failed to produce extra seeds. Particularly in the *mfs1* mutant, some spikelets also formed two paleae and lemmas, suggesting that these spikelets were prone to produce two florets. In Ehrhartoideae, Ehrharta has one terminal floret and two lateral florets that only contained two unreduced lemmas inside a pair of large glumes (Kellogg, 2009; Lin *et al*., 2014). In contrast, the *df1‐1* spikelets contained two florets: a terminal floret and a secondary floret, which consisted of four whorls of floral organs and produced two seeds within each spikelet. Moreover, the spikelet meristem is indeterminate and also induces two to six florets in wheat, which results in the formation of more seeds within a single spikelet. These findings revealed that the presence of two or three florets within a single spikelet is possible in the genus *Oryza*.

A series of genes related to panicle branching have been cloned, such as *DEP1*,* GN1a/OsCKX2*,* IPA1* and *NAL1*, which mainly improve rice yield by increasing the number of panicle branches and controlling the arrangement of spikelets. However, rice breeders have not focused on the number of florets because the spikelets of *Oryzeae* produce a fixed number of florets, implying that the spikelet meristem is determinate. Our findings revealed that *DF1* plays a key role in the regulation of spikelet determinacy. Inducing a switch to indeterminacy in the spikelet meristem with mutated *DF1* or prolonging the activity of the spikelet meristem with mutated *DF1*,* MFS1*,* TOB* and *SNB* may provide a new means to develop rice cultivars with multiflorets spikelet for increasing grain number per panicle. According to the present study, it is possible to breed a multiflorets spikelet by designing and mining those genes which regulate the determinacy and indeterminacy of the spikelet meristem in rice. In summary, two‐/three‐floret spikelet as a novel resource has the potential to further increase rice yield.
